# Systematic Review: Are the Elderly With Diabetes Mellitus Type 2 Prone to Fragility Fractures?

**DOI:** 10.7759/cureus.14514

**Published:** 2021-04-16

**Authors:** Ioannis Papaioannou, Georgia Pantazidou, Zinon Kokkalis, Neoklis Georgopoulos, Eleni Jelastopulu

**Affiliations:** 1 Orthopedics, General Hospital of Patras, Patras, GRC; 2 Otolaryngology - Head and Neck Surgery, General Hospital of Patras, Patras, GRC; 3 Orthopedics, Patras University Hospital, Patras, GRC; 4 Endocrinology, Patras Univeristy Hospital, Patras, GRC; 5 Department of Public Health, University of Patras, Patras, GRC

**Keywords:** elderly, fragility fractures, diabetes type 2

## Abstract

Diabetes mellitus type 2 (T2DM) is an emerging public health issue with high prevalence rates among older adults while fragility fractures constitute a significant public health burden with a great impact. Osteoporosis is the most important metabolic bone disease in patients with diabetes mellitus. Based on current evidence, individuals with T2DM are more vulnerable to fragility fractures than their non-diabetic counterparts, although until now, there aren’t any systematic reviews or meta-analyses concerning the impact of T2DM on the risk of fragility fractures in elderly patients. The aim of this study is to fill this gap in the current literature concerning this specific patient group.

Literature in PubMed and Google Scholar was searched for relevant articles published up to January 2021. The keywords used were: elderly, diabetes mellitus type 2, and fragility fractures. Among the 180 articles retrieved, only four full-text articles were eligible and, finally, two studies (one population-based cohort study and one cross-sectional study) met the inclusion criteria for the review. Although we identified 15 records through the manual research, finally 17 records were included in the current review. The records retrieved from the manual research were 11 prospective cohort studies, two population-based studies, one prospective observational study, and one retrospective cohort study. The author's name, year of publication, country, type of study, and number of patients were reported.

According to this systematic review, there is almost consensus about the increased prevalence of all kinds of fragility fractures and especially low-energy hip fractures among elderly patients with T2DM compared with their counterparts without T2DM while there is relative controversy concerning non-vertebral fractures. Vertebral fractures in the elderly with T2DM require further evaluation because the results from cohort studies are more conflicting. Finally, insulin usage can increase the possibility of fragility fractures and can even double this risk. Bone fragility should be recognized as a new complication of T2DM, especially in elderly patients, due to several additional aggravating factors such as senile osteoporosis, severe vitamin D deficiency, presence of many comorbidities, increased possibility of insulin usage, and the presence of diabetes-related complications (mainly neuropathy and retinopathy). Clinicians who treat these patients should be aware of the special diagnostic and therapeutic approaches concerning these patients.

## Introduction and background

Diabetes mellitus type 2 (T2DM) is an emerging public health issue with a high prevalence among older adults (>60 years old). The main causes are increased life expectancy and lifestyle alterations. It is estimated that 20% of the elderly worldwide suffer from T2DM while a similar proportion has undiagnosed T2DM [1]. On the other hand, fragility fractures constitute a significant public health problem with a great impact. Globally, one in three women and one in five men will experience a fragility fracture resulting in a hospital visit every three seconds [2]. More than 250,000 hip fractures occur annually in older adults in the USA [3]. This tremendous public health threat results in high human and socio-economic burden, morbidity, mortality, and increased health costs. All fragility fractures, such as hip, vertebral, and non-vertebral fractures, are accompanied by significant morbidity and increased risk for future fracture. Osteoporosis is the most important metabolic bone disease in patients with diabetes mellitus. In the last decade, there is evidence that individuals with T2DM are more vulnerable to fragility fractures than their non-diabetic counterparts [4-5], although until now no review or meta-analysis concerning the impact of T2DM on the risk for fragility fractures in elderly patients has been conducted. The aim of this study is to fill this gap in the current literature concerning this specific patient group.

## Review

Eligibility was determined according to the Population, Intervention, Comparison, Outcomes, and Study (PICOS) criteria.

Type of study

All articles in the English language, assessing the correlation of fragility fractures and diabetes mellitus type 2 (T2DM) in the elderly population and especially in those over 60 years of age up to January 2021 were eligible. Studies were eligible if they were longitudinal (prospective or retrospective studies, prospective cohort, case-control, cross-sectional, or randomized control studies) and if they reported the association of T2DM and fragility fractures (any of them, such as hip, vertebral, or non-vertebral fragility fractures) in this specific age group over 60 years old. We complemented the search by the manual scanning of reference lists of identified articles. The search was limited to studies conducted in humans.

Exclusion criteria

Studies with subjects with a mean age below 60 years were excluded from this review. Case series, case reports, animal studies, and studies published in languages other than English were also excluded.

Type of participants

Elderly (>60 years old) patients with T2DM and fragility fractures participated. The research was up to January 2021.

Types of outcome measures

We included studies if they measured the following: correlation of the risk of fragility fractures in elderly patients over 60 years old with established type 2 diabetes mellitus or newly diagnosed T2DM. The fragility fractures concerned all types of low-energy fractures, including hip fractures, vertebral fractures, and non-vertebral fractures. The non-vertebral fractures included proximal humerus, foot, ankle, and distal radius fractures.

Information sources and search methods

A systematic manual search was conducted in PubMed (including ahead of print and Epub) and the first 100 articles of Google Scholar databases published up to January 2021 were selected. The keywords used were the following: elderly, diabetes mellitus type 2, and fragility fractures. Among the 180 articles retrieved, only four full texts were eligible and, finally, two studies (one population-based cohort study and one cross-sectional study) met the inclusion criteria for the review. Although we identified 15 records through the manual research, finally 17 records were included in the current review. Records retrieved from the manual research were 11 prospective cohort studies, two population-based studies, one prospective observational study, and one retrospective cohort study. The author's name, year of publication, country, type of study, and number of patients were reported. Titles and abstracts were screened by the authors, full texts were retrieved for the manuscripts found relevant for the topic, and clearly irrelevant articles were excluded. The retrieved full-text articles were assessed for eligibility for inclusion and data were extracted by the authors. Any disagreement in the selection of articles and data was solved by consensus.

**Figure 1:**
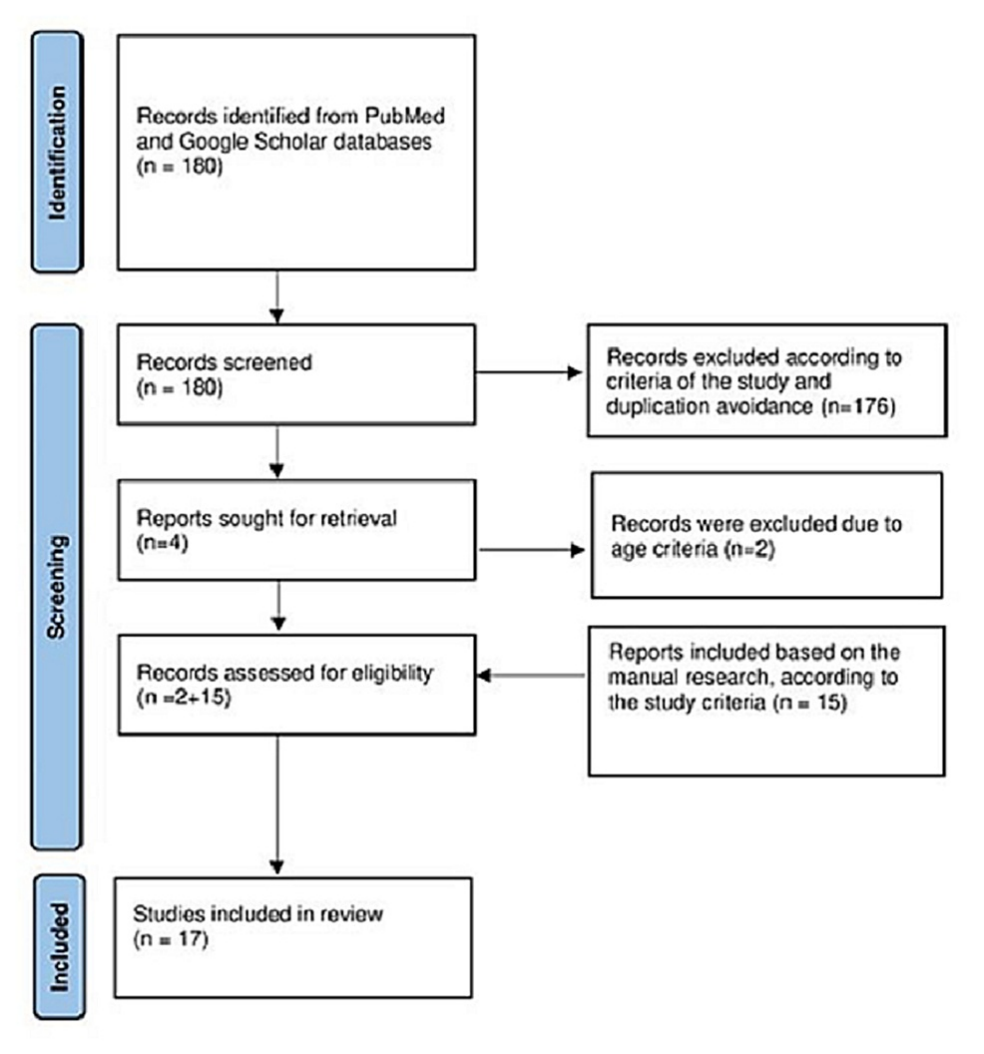
The different phases of the literature research

The different phases of the literature search are illustrated in Figure 1.

For the assessment of the study quality, the risk of bias assessment according to Newcastle-Ottawa was applied (Table 1).

**Table 1:** Quality of the selected studies by using the Newcastle-Ottawa scale

Author	Selection	Compatibility and outcome	Total score
Schwartz et al [6]	4	4	8
Ottenbacher et al [7]	4	4	8
Taylor et al [8]	4	4	8
Strotmeyer et al [9]	4	4	8
Gerdhem et al [10]	4	3	7
Dobnig et al [11]	4	4	8
Bonds et al [12]	4	4	8
Sosa et al [13]	4	4	8
Schwartz et al [14]	4	4	8
Reyes et al [15]	4	3	7
Napoli et al [16]	4	4	8
Martinez-Laguna et al [17]	4	3	7
Lee et al [18]	4	4	8
Looker et al [19]	4	4	8
Napoli et al [20]	4	4	8
Goldshtein et al [21]	4	3	7
Guo et al [22]	4	3	7

Results

A total of 180 studies were identified through the database search (PubMed and Google Scholar). Among the 180 articles retrieved, only four full texts were eligible and, finally, two studies met the inclusion criteria for the review. Although we identified 15 records through the manual research, finally 17 records were included in the current review. The majority of the articles were prospective cohort studies (11 out of 17 studies), one retrospective cohort study, one cross-sectional study, one prospective observational study, and three population-based cohort studies. Eight (8) studies were conducted in the USA, three (3) in Spain, two (2) in Italy, and one (1) in Sweden, Austria, China, and Israel. A total of 603,995 patients were included in the review. Five studies included only elderly women (total 111,461 participants), three studies only elderly men (totally 198,159), and the remaining nine studies included both men and women (total 305,625 participants).

The majority of studies (13 out of the total 17 studies) conclude that elderly patients with T2DM are at increased risk for fragility fractures. Only four studies didn’t manage to correlate T2DM with a higher incidence of fragility fractures in this specific group of patients. Taken into consideration the large number of participants in this review (603,995), it is worth noting that barely 9,000 subjects (specifically 8,992 participants) have been included in the four studies, which support the thesis that T2DM doesn’t increase the risk for fragility fractures in elderly patients. To realize the extremely small number of participants in these four studies, the percentage compared to the total number of included subjects to this review is only 1.48%.

Eight out of the 17 studies of this review include all possible types of fragility fractures without specifying the exact fractured bone. These eight studies are one retrospective cohort, four prospective cohorts, one prospective observational study, one population-based cohort, and one cross-sectional study. All the above-mentioned studies, except for the retrospective cohort study managed to strongly correlate fragility fractures (in general) with type 2 diabetes mellitus in the elderly population. Participants in the studies, which support the thesis that elderly with T2DM are at increased risk for any fragility fracture were 242,482 while the subjects of the retrospective cohort, which concludes that T2DM doesn’t add any risk for fragility fractures in this specific age group were only 1,132.

The possible correlation of hip fractures and T2DM in elderly patients has been evaluated in seven studies (five prospective cohort studies and two population-based cohort studies), which included 379,293 participants. Five studies, three prospective cohorts, and two population-based cohort studies with 377,427 participants conclude that elderly patients with T2DM are at increased risk for low-energy hip fractures. Only two prospective cohort studies with 1,866 participants support the thesis that T2DM does not increase the risk for hip fractures among elderly patients.

As far as vertebral fractures are concerned, the results are different compared with those of hip and fragility fractures in general. We included two studies that specify risk for vertebral fractures in elderly patients with T2DM. These studies are two prospective cohorts with 6,196 participants and both of them conclude that T2DM is not associated with a higher prevalence or incidence of vertebral fracture in the elderly, even after adjustment of body mass index (BMI) and bone mineral density (BMD).

Non-vertebral fractures have been evaluated in four studies of this review. All these studies are prospective cohorts with 17,514 participants. The results of non-vertebral fractures are controversial, as two studies (with 15,648 participants; 89.45%) support that T2DM is a risk factor for non-vertebral fractures, such as proximal humerus, distal radius, and foot fractures in elderly patients, while the other two papers (with 1,866 participants, 10.65%) conclude that T2DM does not increase the risk for non-vertebral fractures in elderly patients.

It is worth noting that two studies (both prospective cohorts) of this review with 8,878 participants highlight the thesis that insulin usage can increase the possibility of fragility fractures and can even double this risk while another prospective cohort study notes that pre-diabetes is not significantly associated with higher fracture risk.

Table 2 summarizes the correlations between T2DM and fragility fractures in the elderly population (Table 2).

**Table 2:** Correlations and results between diabetes mellitus type 2 and fragility fractures in the elderly population Cohorts with bold type consist of the unique four studies that support that T2DM is not an aggravating factor for the skeletal health of the elderly. T2DM: type 2 diabetes mellitus, BMD: bone mineral density, BMI: body mass index

Author	Year	Country	Study type	Patients-Gender	Results
Schwartz et al [6]	2001	USA	Prospective cohort study	9.654- Female	T2DM is a risk factor for hip, proximal humerus, and foot fractures among older women, suggesting that fracture prevention efforts should be a consideration in the treatment of diabetes.
Ottenbacher et al [7]	2002	USA	Prospective cohort study	2.884- Male and Female	T2DM was associated with an increased risk for a hip fracture in older Mexican Americans, particularly subjects taking insulin.
Taylor et al [8]	2004	USA	Prospective cohort study	6.787- Female	Clinicians should be alert to factors (including T2DM) other than BMD that place older women at a high risk of hip fracture.
Strotmeyer et al [9]	2005	USA	Prospective cohort study	3.075- Male and Female	These results indicate that older white and black adults with T2DM are at higher fracture risk compared with nondiabetic adults with a similar BMD.
Gerdhem et al [10]	2005	Sweden	Retrospective cohort study	1.132- Female	Women with T2DM had no more lifetime fractures than women without the diabetic disease.
Dobnig et al [11]	2006	Austria	Prospective cohort study	1.664- Male and Female	T2DM does not increase the risk for hip or other non-vertebral fractures in nursing home patients.
Bonds et al [12]	2006	USA	Prospective cohort study	93.676-Female	Women with type 2 diabetes are at increased risk for fractures.
Sosa et al [13]	2009	Spain	Prospective cohort study	202-Female	The prevalence of vertebral, hip, and non-vertebral fractures did not increase in type 2 DM.
Schwartz et al [14]	2011	USA	Prospective observational study	16.885- Male and Female	The fracture risk was higher for diabetic patients for a given T-score and age or for a given FRAX score.
Reyes et al [15]	2014	Spain	Population-based cohort study	186.171-Male	Common co-morbidities including diabetes are independently associated with an increased risk of hip fracture in elderly men.
Napoli et al [16]	2014	Italy	Prospective cohort study	5.994-Male	The risk of non-vertebral fracture is 30% higher in men with diabetes for a given BMD. Men who take insulin have more than double the risk of fractures.
Martinez-Laguna et al [17]	2014	Spain	Population-based cohort study	171.931- Male and Female	Newly diagnosed T2DM patients are at a 20% increased risk of hip fracture even in the early stages of the disease.
Lee et al [18]	2015	USA	Prospective cohort study	2704- Male and Female	The current study shows that older women with T2DM have a significantly greater risk of incident fracture than those without T2DM.
Looker et al [19]	2016	USA	Prospective cohort study	4588- Male and Female	The diabetes–fracture relationship was stronger in Mexican Americans and non-Hispanic blacks. Pre-diabetes was not significantly associated with higher fracture risk.
Napoli et al [20]	2018	Italy	Prospective cohort study	5994-Male	T2DM was not associated with a higher prevalence or incidence of vertebral fracture in older men, even after adjustment for BMI and BMD.
Goldshtein et al [21]	2018	Israel	Population-based cohort study	87.224- Male and Female	This study confirms the higher fracture risk of osteoporotic patients with T2DM, as compared to osteoporotic patients without T2DM.
Guo et al [22]	2020	China	Cross-sectional study	3430- Male and Female	Patients with T2DM have a higher risk for fractures even when they have sufficient BMD.

According to this review, there is adequate evidence to support the correlation of T2DM with the increased risk of fragility fractures in the elderly. Among the studies included in this review, only four papers with 1.48% of the total number of participants state that T2DM does not constitute a considerable risk factor for fragility fractures in the elderly population. According to this review, there is almost consensus about the increased prevalence of all kinds of fragility fractures and especially hip fractures among elderly patients with T2DM compared with their counterparts without T2DM. There is relative controversy concerning non-vertebral fractures. These fractures have been evaluated in four studies in this review, and the results are conflicting although the participants included in the two studies that support that T2DM increases the risk for non-vertebral fractures are the vast majority (89.45%). The results of vertebral fractures in elderly patients with T2DM differ significantly. The two studies that have evaluated vertebral fractures in this specific patient group conclude that T2DM is not associated with a higher prevalence or incidence of vertebral fractures. Although we should take into consideration the fact that the studies that support that the elderly with T2DM are at increased risk for fragility fractures include vertebral fractures also. These studies count much more participants than the two studies that evaluated the risk for vertebral fractures only (242,482 and 6,196, respectively).

Discussion

The increased fracture risk in patients with type 2 diabetes mellitus is multifactorial. T2DM adversely affects the bones with many pathophysiological mechanisms, although the complications of the disease and the adverse effects of the pharmacological treatment of T2DM also play a crucial role in bone fragility in these patients and especially the elderly [23].

Hyperglycemia has many implications for the bone, as it promotes the accumulation of advanced glycosylation end products (AGEs) in the collagen fibers, which alter the structure and strength of bone. AGEs accumulation promotes the synthesis of interleukin 6 (IL-6), which reduces osteoblast production and their differentiation and mineralization. In addition, IL-6 inhibits collagen type 1 synthesis and increases osteoclast-induced bone resorption resulting in bone fragility. AGEs and low insulin levels cause a reduction in insulin-like growth factor I (IGF-I) secretion, which consists of a bone anabolic protein. Furthermore, pancreas dysfunction leads to reduced levels of amylin, which stimulates the proliferation of osteoblasts [23-24].

T2DM is strongly correlated with oxidative stress, increased reactive oxygen species production (ROS), and acceleration of inflammatory cytokines such as tumor necrosis factor (TNF) and IL-6. We analyzed the role of IL-6 in bones while TNF promotes osteoclastogenesis and inhibits osteoblastogenesis. On the other hand, ROS impacts the differentiation and survival of osteoblasts, osteoclasts, and osteocytes [24].

Brown adipose tissue is reduced inT2DM. This tissue secretes insulin-like growth factor-binding protein 2 (ILGF-BP2) and Wnt10b, which are both bone anabolic factors and promote osteoblastogenesis. Another mechanism that causes bone fragility in T2DM patients is marrow fatty infiltration. Adipose tissue produces free fatty acids and ROS in bone marrow, which both are a hazard for osteoblast function and induce osteoblasts apoptosis [23].

Hyperglycemia causes osmotic diuresis accompanied by hypomagnesemia, which adversely affects parathyroid hormone (PTH) secretion. The reduced PTH levels and hypercalciuria, due to kidney dysfunction, result in a negative calcium balance and severe bone mineralization disorder [24].

Finally, T2DM is strongly correlated with the loss of the incretin effect due to a reduction in gastric inhibitory polypeptide (GIP) and glucagon-like peptide 1 (GLP1) secretion from the gut [24]. These two hormones are called incretins and promote osteoblastogenesis via the stimulation of mesenchymal stem cell proliferation. Loss of incretin effect accompanied with increased sclerostin secretion from osteocytes [25] inactivate the Wnt pathway, which inhibits bone formation, decreases bone turnover, and finally leads to osteopenic state.

Bone turnover is decreased in T2DM patients while lower levels of bone formation and absorption biochemical markers have been reported by many studies to T2DM patients compared with non-diabetic counterparts [26]. T2DM patients fractured more frequently because they cannot repair micro-cracks of the bone due to inadequate bone turnover. The healing process requires osteoclastic resorption to remove the damage and osteoblastic formation to replace the resorbed bone. Increased cortical porosity contributes also to the increased prevalence of fractures in T2DM patients [27].

There is a paradox worth noting in patients with T2DM. Several epidemiological studies highlight the fact that T2DM is associated with an increased risk of fractures although both men and women with T2DM typically have normal to high bone mineral density (BMD) compared with their age-adjusted non-diabetic counterparts. Increased body mass index (BMI) affects BMD measurements and individuals with higher weight such as those with T2DM have higher BMD compared to their age-matched counterparts [28]. Based on this paradox, many studies conclude that bone mineral density is not sensitive enough to assess the risk for fragility fractures in type 2 diabetic patients [29].

The established risk factors for fractures in patients with T2DM are the following: longstanding diabetes mellitus, presence of diabetes mellitus complications and especially diabetic retinopathy, history of insulin use, and poor glycemic control [30].

The elderly patients with T2DM have many additional predisposing factors for increased fracture prevalence. These factors are the presence of senile osteoporosis, more severe vitamin D deficiency [31], comorbidities and pharmacotherapies that affect bone quality and predispose to falls, longstanding diabetes-induced bone fragility [30], and increased probability for diabetes-induced complications such as retinopathy, diabetic peripheral neuropathy, and poor cognitive performance, which increase the risk of a fall and subsequent fracture [32].

Many authors suggest that skeletal fragility should be included in the chronic complications of T2DM and this disease should be considered among the causes of endocrine osteoporosis [33]. Many studies correlate the increased fracture risk in diabetic patients due to microvascular disease and the subsequent limited vascular supply to the skeleton and specifically in cortical bone [24]. The impaired vascular supply to cortical bones in T2DM patients may be the cause of cortical porosity in these patients compromising the proper bone formation [24]. Cortical porosity is significantly higher in diabetic subjects and especially in those with fragility fractures [34]. This finding has been proved via the high-resolution peripheral quantitative computed tomography (HR-pQCT). Larger cortical pores impair bone strength and increase the fracture risk of patients with type-2 diabetes [34]. This finding consists of the mechanical aspect of diabetes-induced bone fragility.

BDM is an inadequate determinant of fracture risk in patients with T2DM while until now the Fracture Risk Assessment Tool (FRAX) score underestimates the fracture probability in these patients, suggesting that future fracture prediction algorithms should consider diabetes as an independent risk factor [35]. Unfortunately, BMD can’t assess changes in bone quality of these patients. Patients with T2DM have relatively increased BMI and this fact impacts higher BMD in the dual-energy X-ray absorptiometry (DEXA) scan [24]. So, diabetic patients present a paradox worth noting; they usually have high BMD and simultaneously increased propensity to fractures. The appropriate estimation of fracture risk in patients with T2DM is difficult, although clinicians should be aware of some additional diagnostic tools. First, trabecular bone score (TBS) is a new texture parameter that analyzes pixel gray-level variations in the DEXA image and consists of an indirect bone micro-architecture index. TBS is a specific measurement of DEXA, which is often reduced in T2DM patients, although it requires additional software [36]. TBS is inversely correlated with fasting plasma glucose levels, fasting insulin, and hemoglobin A1c (HbA1c) [37]. The quantitative computed tomography (QCT) assesses noninvasively the bone micro-architecture at the distal radius and tibia while the HR-pQCT measures the volumetric BMD (vBMD) and estimates the mechanical parameters of bone. T2DM is strongly correlated with lower vBMD and higher cortical porosity [35]. The increased vBMD of trabecular bone in patients with T2DM is accompanied by increased cortical porosity, mainly at peripheral skeletal sites [35]. Microindentation is a more invasive technique, which assesses the bone material strength index (BMSi) via probe insertion into the cortical bone’s surface (anterior tibia), which causes microscopic fractures [37]. T2DM patients have significantly lower BMSi as compared to their non-diabetic counterparts. Poor glycemic control in T2DM subjects is associated with lower BMSi. Bone biopsy, the so-called bone histomorphometric analysis, consists of an invasive technique for the assessment of bone remodeling rates, which measures the bone volume, osteoid volume, osteoid thickness, cortical thickness, and osteoblast surface [38]. Bone turnover markers is a well-known assessment method for bone remodeling, although in T2DM, both bone formation and absorption biomarkers are reduced. C-terminal telopeptide of type 1 collagen (CTX-1), osteocalcin (OC), N-telopeptide of type 1 collagen (NTX-1), procollagen type 1 N-terminal propeptide (P1NP), and tartrate-resistant acid phosphatase (TRAP) are reduced in T2DM patients [36]. Parathyroid hormone (PTH) levels are much lower (up to 50%) in T2DM subjects as compared to their non-diabetic counterparts. It is worth noting that elevated sclerostin levels [36] and both serum and urine pentosidine [39] have been also associated with increased fracture risk in T2DM.

Pharmacological treatment of T2DM should balance the drug-induced skeletal fragility and their ability to control the glycemic status of the patient. Clinicians should be aware of the impact of these pharmacological agents on bone strength (BMD) and their contribution to fracture risk. Glucagon-like peptide 1 (GLP-1) agonists and metformin have a positive impact on the BMD of T2DM patients while these drugs reduce also fracture risk. On the other hand, sulfonylureas, glitazones, canagliflozin, and insulin have a negative impact on BMD and increase fracture risk. Incretin mimetic drugs and SGLT2 inhibitors (except canagliflozin) have a neutral impact on both BMD and fracture risk [40].

According to this systematic review, there is adequate evidence to support the correlation of T2DM with the increased risk of fragility fractures in the elderly. So, clinicians who treat these patients should also address bone fragility according to the specific modifications that accompany T2DM patients. First, the specific fracture risk factors for patients with T2DM should be taken into consideration. These are the duration of diabetes over five years, HbA1c over 7%, consumption of anti-diabetic drugs that affect bone strength and increase fracture risks, such as insulin, canagliflozin, glitazones, and sulfonylureas, and the presence of diabetes complications, especially peripheral neuropathy, artery disease, retinopathy, and nephropathy. According to the International Osteoporosis Foundation (IOF), diabetes and bone workout group, T-score ≤ -2 and not -2.5 is a strong indication of osteoporosis treatment administration [40]. DEXA should be accompanied by TBS measurement while the FRAX score should be adjusted for T2DM [40-41]. This should be done by clicking the choice of secondary osteoporosis in the FRAX tool. Concerning the treatment of osteoporosis in patients with T2DM, there is a great lack of evidence about the effectiveness (impact on BMD and fracture risk) of existing anti-osteoporotic treatments in patients with T2DM. Current evidence supports that alendronate, risedronate, and teriparatide have a positive impact on BMD in these patients, although there is a lack of evidence concerning the remaining anti-osteoporotic agents such as zoledronate, ibandronate, raloxifene, and denosumab [42].

## Conclusions

In the last 20 years, there has been a tremendous increase in understanding diabetes mellitus type 2-induced bone fragility. The elderly (over 60 years old) patients with T2DM are a specific, frail, and fragile group that requires further investigation and monitoring. According to this systematic review, there is almost consensus about the increased prevalence of all kinds of fragility fractures and especially hip fractures among elderly patients with T2DM as compared with their counterparts without T2DM while there is relative controversy concerning the non-vertebral fractures. Vertebral fractures in the elderly with T2DM require further evaluation because the results of cohort studies are more conflicting. Finally, insulin usage can increase the possibility of fragility fractures and can even double this risk. Bone fragility should be recognized as a new complication of T2DM, especially in elderly patients. The elderly are even more vulnerable to T2DM-induced bone fragility due to several additional aggravating factors, which include senile osteoporosis, severe vitamin D deficiency, presence of many comorbidities, increased possibility of insulin usage, presence of diabetes-related complications, and especially diabetic neuropathy and retinopathy, which predispose to increased falls. These specific features of this patient group should absolutely be taken into consideration. Clinicians who treat these patients should be also aware of the special diagnostic and therapeutic approaches concerning these patients.
